# Threats across boundaries: the spread of ESBL-positive *Enterobacteriaceae* bacteria and its challenge to the “one health” concept

**DOI:** 10.3389/fmicb.2025.1496716

**Published:** 2025-02-21

**Authors:** Shaqiu Zhang, Jing Yang, Muhammad Abbas, Qian Yang, Qianlong Li, Mafeng Liu, Dekang Zhu, Mingshu Wang, Bin Tian, Anchun Cheng

**Affiliations:** ^1^Avian Disease Research Center, College of Veterinary Medicine, Sichuan Agricultural University, Chengdu, China; ^2^Key Laboratory of Animal Disease and Human Health of Sichuan Province, Sichuan Agricultural University, Chengdu, China; ^3^Engineering Research Center of Southwest Animal Disease Prevention and Control Technology, Ministry of Education of the People's Republic of China, Chengdu, China; ^4^International Joint Research Center for Animal Disease Prevention and Control of Sichuan Province, Sichuan Agricultural University, Chengdu, China; ^5^School of Veterinary Medicine, University of Surrey, Guildford, United Kingdom

**Keywords:** antimicrobial resistance, *Enterobacteriaceae*, extended-spectrum *β*-lactamases, mobile genetic elements, horizontal gene transfer, plasmid incompatibility groups

## Abstract

*β*-lactam antibiotics are essential medications for treating human diseases. The spread of extended-spectrum β-lactamase-producing *Enterobacteriaceae* (ESBL-PE) exists globally in multiple reservoirs and the natural environment and poses an immense threat to public health. Plasmid incompatibility groups, such as IncFIA, IncI1, IncY, IncFIB, IncN, IncFIC, IncX4, IncB/O/K/Z, IncHI1/2, and IncA/C, which exist in humans, animals, and the environment, carrying *bla*_CTX-M_, *bla*_TEM_, and *bla*_SHV_ genes. The IS*Ecp1* upstream and orf477 downstream of *bla*_CTX-M_ genes, as well as other mobile genetic elements (MGEs) such as IS*903* and IS*26*, are involved in capturing and mobilizing antibiotic-resistant genes (ARGs). The *bla*_CTX-M-15_ gene is the most common among all discussed reservoirs. The environmental reservoir and propagation mode of ESBL-PE are increasing and difficult to control. The reasons include but are not limited to bacterial adaptability and horizontal gene transfer (HGT) mediated by MGEs and plasmids. Conjugation is a pathway of HGT that is almost uncontrollable. MGEs and plasmids such as Tn*3*, IS*1380* families, IncI1, IncK, and IncN are facilitating HGT of *bla*_CTX-M_ genes. This review highlights the need to monitor trends in antimicrobial resistance (AMR) in the natural environment. Therefore, policies such as antibiotic management plans, training for healthcare providers and/or patients, cautious use of antibiotics, the need for epidemiological networks, pre-travel consultations, World Health Organization (WHO) infection control and biosafety guidelines, and other intervention measures are considered desirable.

## 1 Introduction

ESBL-PE, first reported in Germany in 1983 (Paterson and Bonomo, 2005), are present globally in humans, animals, and the environment, and have been spreading particularly rapidly in the last two decades (Pitout and Laupland, 2008; Lee et al., 2020). ESBL-PE is a community-level problem in many regions, such as Japan, China, France, Vietnam, and the Netherlands, as well as in low-or-middle-income countries (Castillo-Tokumori et al., 2017; Flateau et al., 2018; Arcilla et al., 2019; Nguyen V. T. et al., 2019; Kwok et al., 2020; Oka et al., 2022). In 2022, the European Antimicrobial Resistance Surveillance Network (EARS-Net) collected AMR data for the eight bacterial species from 30 European countries. Of the eight species, *Escherichia coli* (*E. coli*) was shown to be the most prolific, followed by *Staphylococcus aureus* (*S. aureus*) and *Klebsiella pneumoniae* (*K. pneumoniae*) (European Centre for Disease Prevention and Control, 2020). In the same year, the China Antimicrobial Resistance Surveillance System received surveillance data from 1997 hospitals in China, and *E. coli* was the most common Gram-negative bacterium, followed by *K. pneumoniae* and *Pseudomonas aeruginosa* (*P. aeruginosa*), and 46.8% of *E. coli* were resistant to third-generation cephalosporins (Moran et al., 2023). ESBL-PE often affects US inpatients, with approximately 290,220 people affected per year (Gupta et al., 2019).

AMR poses a significant public health threat and is a key issue within the “One Health” approach. The emergence of ESBL-PE, a bacteria found in animals, humans, and the environment, exemplifies this concept (Perestrelo et al., 2023). The term “One Health” emerged in 2003–2004 during the SARS and H5N1 outbreaks, emphasizing the need for interdisciplinary action against diseases (Atlas, 2013). The COVID-19 pandemic underscores the interdependence of human, animal, and environmental health, which is also crucial for addressing AMR. ARGs in these hosts contribute to AMR, posing a pandemic risk (Despotovic et al., 2023). It is vital to integrate human, animal, and environmental health into the “One Health” framework to effectively tackle the growing challenge of AMR (Muloi et al., 2023).

We flagged *E. coli* as one of the main threats to public health and environmental safety, and its contribution to the spread of ARGs is linked with integrons, transposons, and transmissible plasmids (Zhang et al., 2020). Plasmids, as the core of ARG transmission within the “One Health” framework, are the foundation for the spread of antibiotic resistance among bacteria and within and between habitats (Castañeda-Barba et al., 2024). Plasmid-mediated colistin, carbapenem, and tigecycline resistance genes, accompanied by ESBL genes, represent a potential public health concern (Clemente et al., 2019; Yang et al., 2023). Further, we have recently reviewed the co-existence of ESBL genes with resistance to colistin and carbapenem (Zhang et al., 2021). The spread of ESBL genes in natural environments and in humans is a burning issue, because plasmid-mediated HGT transforms non-resistant bacterium into resistant ones (Kawamura et al., 2017). Insertion sequences (IS) such as IS*Ecp1*, IS*903*, IS*3*, IS*1380* families, and *orf*477 have been linked with the genetic environment of ESBL genes (Lifshitz-Ziv et al., 2018; Valizadeh et al., 2021; Awosile and Agbaje, 2021; Sultan et al., 2022). Furthermore, MGEs and plasmids like Tn*3*, IncI1, IncK, IncFII, and IncN are enhancing the spread of these genes in ESBL-PE (Maamar et al., 2016; Lifshitz-Ziv et al., 2018; Sultan et al., 2020; Berbers et al., 2023). Routes of transmission of ESBL-PE include but are not limited to hospital infections (Artero et al., 2017), fecal carriage (Tamta et al., 2020), travelers (Nakayama et al., 2019), agriculture (Richter et al., 2020), wastewaters (Li et al., 2016), poultry production (Tansawai et al., 2019), and wild animals (Fuentes-Castillo et al., 2023). Researchers recognize the ESBL-PE spread pathways are associated with direct contact with animals, a poor living environment, and handling of contaminated meat/carcasses. In addition, flows out of hospitals, wastewater treatment plants (WWTPs) in agricultural fields, and the natural environment represent dangers.

**Figure 1:**
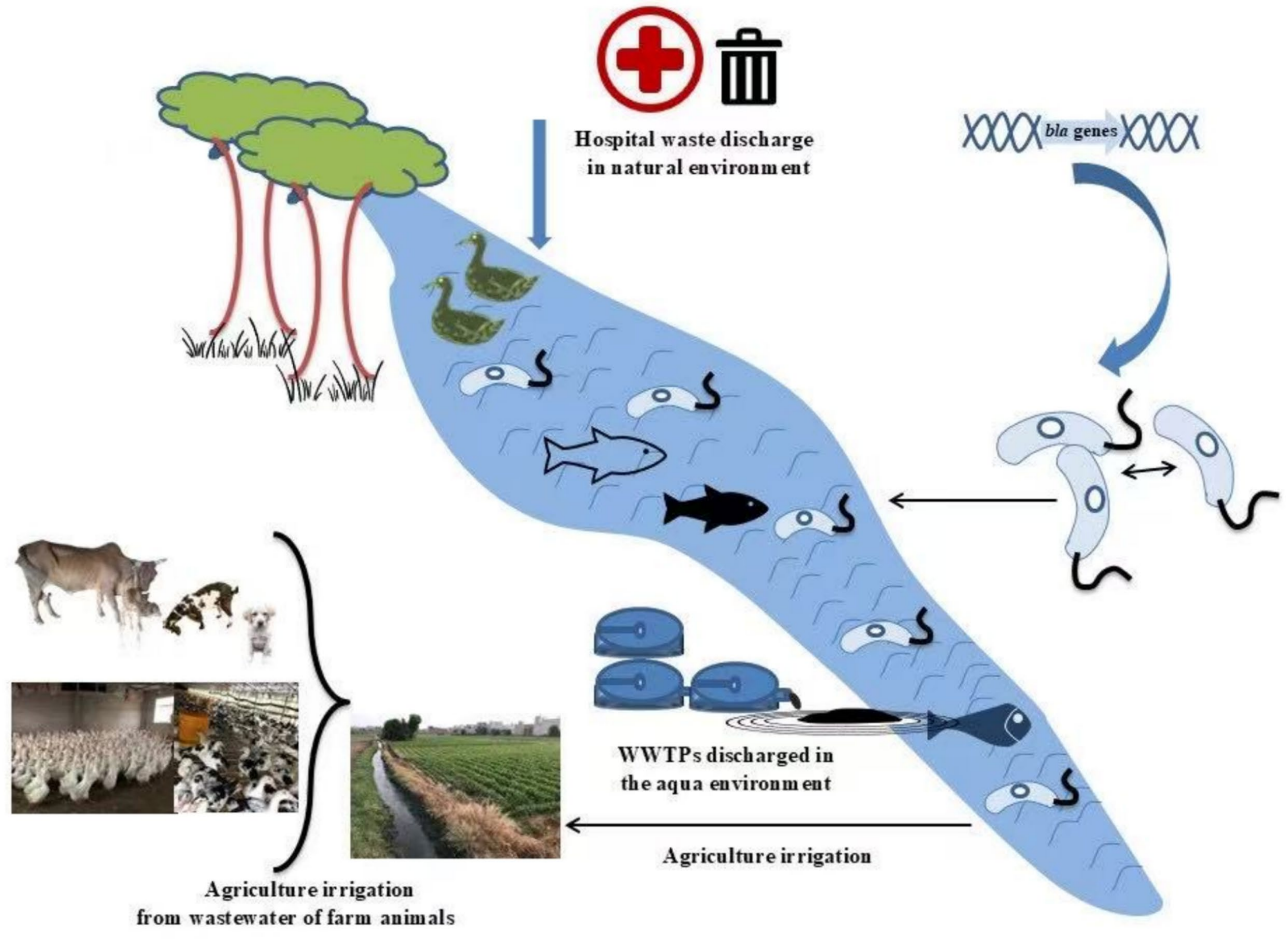
The illustration illustrates the release of ESBL-PE into the environment via hospital waste and WWTPs and their transmission between humans, animals, and the natural environment. These bacteria carry ARGs that may be passed between different hosts via plasmids and MGEs. The figure also shows how agricultural irrigation may facilitate the spread of these ARGs. This process highlights the complexity of transmission across species and environments and emphasizes the importance of implementing “One Health” strategies to monitor and control antibiotic resistance.

As depicted in Figure 1, ESBL genes evolve and spread dramatically in environmental settings through transmissible plasmids, transposons, and MGEs, compromising safety and public health. This framework is concluded during this organized review, and the objective was to identify available evidence related to the capture, transmission, and expression mechanisms of ESBL genes and associated risk factors (RFs), plasmids, or MGE-mediated HGT among bacteria that affect animals, humans, and the natural environment. People from developed areas when traveling to underdeveloped infected communities can enhance the spread of ESBL-PE to healthy people and the environment. Lastly, the preventive control and restrictive measures for the ESBL-PE are summarized.

## 2 Literature sources and search strategies

Published documents were accessed through databases such as PubMed, Science Direct, and Google Scholar. Keywords included, but were not limited to, ESBLs, Enterobacteriaceae, risk factors (RFs), risk, infection, travelers, healthcare workers (HCWs), illness, disease, farmworkers (FWs), environment, health care professionals, *bla*_TEM_, *bla*_SHV_, *bla*_CTX-M_, HGT, MGEs, Tn*3*, IS*Ecp1*, antibiotic stewardship programs (ASPs), and occupational health. References cited were searched for additional related studies. The timeframe of the search was limited to English-language literature up to December 31, 2024, in order to ensure that the study incorporated the most recent research findings in the field.

## 3 Plasmid-mediated ESBLs in hospital settings

The spread of ESBL-PE in medical settings has become a major public health issue in recent times (Table 1) (Karlowsky et al., 2022). High diversity in plasmidome in clinical isolates further complicates the situation (Neffe et al., 2022). For example, in Portugal, healthcare students were shown to carry *bla*_CTX-M-1_, as well as *bla*_CTX-M-15_ and *bla*_CTX-M-8_, and most of them are in IncFIA/FIB-type plasmids (Fournier et al., 2020). In Indonesia, *bla*_CTX-M_, *bla*_TEM,_ and *bla*_SHV_, and primarily *bla*_CTX-M-15,_ are circulating among medical students, located on different chromosome regions and plasmids (Rosantia et al., 2020). In Taiwan, *bla*_SHV_ and *bla*_CTX-M_ are predominantly prevalent and associated with IS elements as well as IncA/C-type plasmids (Chao et al., 2023). This may be due to the polluted environment in medical universities or communities.

**Table 1:** Selected studies from hospital settings reporting ESBL-PE in various countries.

Country	Bacteria isolated	Study on	Samples type	No. of samples	Plasmids type	History/ techniques used	Identified genes/genotype/ ESBLs	Years of sampling	Reference
China	*E. coli*	Hospital wards, Environment	Air	NA	NA	Hospital ward environmental samples were examined from various points. PFGE. PCR, MIC, DDST	TEM, SHV, CTX-M	NA	Wu B. et al. (2020), Wu N. et al. (2020)
Spain	*E. coli* *K. pneumoniae*	Health workers	Rectal swabs	258	NA	Six hospitals in Northern Spain were screened. Questionnaire, MLST, PCR	*bla*_CTX-M-15_, *bla*_CTX-M-32_, *bla*_CTX-M-55,_ *bla*_CTX-M-14_, *bla*_CTX-M-27_	NA	Fernández-Verdugo et al. (2020)
Portugal	*E. coli* *K. pneumoniae*	Healthcare students	Fecal	111	IncFIA/FIB, IncFIC, IncP, Inc. I1	Students belonged to 4 years of schooling in bachelors of nursing and/or physiotherapy. PFGE, PCR, PBRT	*bla*_CTX-M-1_, *bla*_CTX-M-15_, *bla*_CTX-M-8_	2018	Fournier et al. (2020)
Germany	*E. coli* *K. pneumoniae*	Hospitalized patients	Fecal	2,971	NA	A prospective cohort study in which the association of ESBL-PE with underlying diseases was determined. PFGE, WGS	NA	2014–2015	Denkel et al. (2020)
Spain	*E. coli* *K. pneumoniae*	Medical and surgical hospital wards	Rectal swabs	10,643	NA	Patients above 18 years old were screened in pneumology, gastroenterology, urology, and neurosurgery units in a university tertiary hospital in Madrid. DDST, PCR	*bla*_CTX-M-15_, *bla*_CTX-M-14_, *bla*_CTX-M-1_, *bla*_CTX-M-9_, *bla*_CTX-M-27_, *bla*_CTX-M-32_, *bla*_CTX-M-55_, *bla*_SHV-2_, *bla*_SHV-12_	2014–2016	Díaz-Agero Pérez et al. (2019)
Uganda	*E. coli* *K. pneumoniae*	Human	Stool	300	NA	Cross-sectional study, the Study population included pastoralist communities with fever, and/or diarrhea without malaria. BD Phoenix 100 automated identification system.	NA	2017	Stanley et al. (2018)
Tanzania	*E. coli* *K. pneumoniae*	Surgical site infections	Rectal swabs, Wound/pus swabs	930	NA	A prospective cohort study to identify ESBL-PE load in surgical patients at the time of admission and discharge. MLST, PCR, DDST	*bla*_CTX-M-15_	NA	Moremi et al. (2018)
Italy	*E. coli* *K. pneumoniae*	Long-term care facility residents	Urine, Rectal, inguinal, oropharyngeal, and nasal swabs	182	NA	The age of Long-term care facility residents was ranging 24–96 years with a median age of 83 years. The women were 56%. PCR	*bla*_CTX-M-15_, *bla*_TEM_, *bla*_SHV_	2016	March et al. (2017)
Italy	*E. coli* *Citrobacter freundii* *Enterobacter agglomerans*	slaughtered pigs and hospitalised patients	faecal samples, urine samples	300	NA	Slaughtered pigs and hospitalised patients. API 20 NE, MIC, PCR, qPCR, WGS	*bla*_CTXM-1_*, bla*_CTX-M-2_*, bla*_TEM-1_, and *bla*_SHV_	2017–2018	Bonardi et al. (2022)

MLST, Multi-locus sequence typing; PFGE, Pulsed-field gel electrophoresis; MIC, Minimum inhibitory concentration; PCR, Polymerase chain reaction; DDST, Double disc synergy test; PBRT, PCR-based replicon typing; WGS, Whole-genome sequencing; NA, Not analyzed/available.

**Figure 2:**
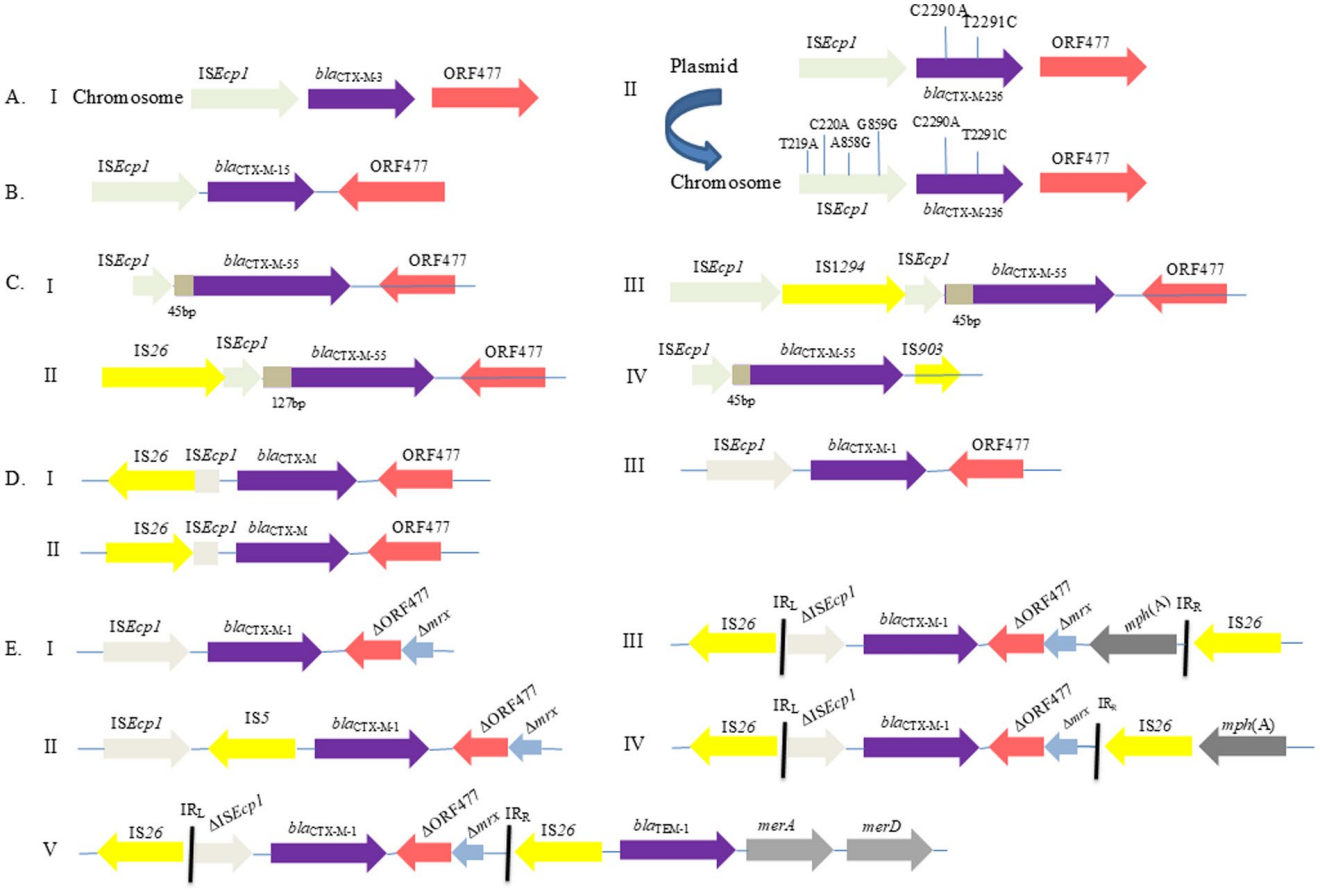
Illustration of the recently reported *bla*_CTX-M_ genetic environment with transposition of IS*Ecp1* identified as a means of dissemination in different reservoirs. **(A)** Genetic environment of a newly identified variant of *bla*_CTX-M-3_ (*bla*_CTX-M-236_) gene; positioned on an IncM2-type plasmid and chromosome with identified allele and loci (Huang et al., 2021). **(B)** Genetic context of *bla*_CTX-M-15_ gene from the urban aquatic environment in India (Singh et al., 2018). **(C)** Four types of clinically oriented genetic environments noted for *bla*_CTX-M-55_ (Hu et al., 2018). **(D)** From the hospital environment, three patterns were noted, *bla*_CTX-M_ genetic environment with IS elements (Valizadeh et al., 2021). **(E)** Schematic presentation of flanking regions of *bla*_CTX-M-1_ genes in plasmids prevalent in healthy humans and food animals (Wang et al., 2015).

Microbial aerosols contain a number of genes, namely *bla*_TEM_, followed by *bla*_CTX-M_ and *bla*_SHV_, in the hospital environment (Wu B. et al., 2020). Interestingly, *bla*_TEM_ is the most common and *bla*_CTX-M_ gene is emerging in *Enterobacteriaceae* in hospitals (Jena et al., 2018; Perera et al., 2022). On environmental surfaces of hospital settings such as in urology, intensive care units, or orthopedic and surgical departments, IS*Ecp1* has been observed largely upstream of *bla*_CTX-M-1_, along with IS*26* in two orientations and downstream of *orf*477 (Figure 2D) (Valizadeh et al., 2021). Isolates from outpatient department, wards, intensive care unit, cabins, and neonatal intensive care units carry the three ESBL genes (Jena et al., 2017). Upon discharge from hospital wards, patients colonize the *bla*_CTX-M-15_ gene at a higher level (Moremi et al., 2018; Díaz-Agero Pérez et al., 2019; Riccio et al., 2021). In addition, a statistically significant relationship between hospital antibiotic use and the prevalence of *bla*_CTX-M-9,_ *bla*_CTX-M-3,_ and *bla*_SHV-5_ was observed in hospital effluents (Korzeniewska and Harnisz, 2013a).

Rehabilitation units in Israel, France, Italy, and Spain revealed that family members (FMs) were more at risk of ESBL-PE acquisition from their family patients than HCWs (Adler et al., 2014; March et al., 2017), which calls for ASPs and infection management strategies.

Approximately 150 million urinary tract infections (UTIs) are diagnosed worldwide annually (Flores-Mireles et al., 2015). A 2017 report showed that the WHO considered *E. coli* to be the main strain causing community- and hospital-acquired UTIs (Ala-Jaakkola et al., 2022). UTIs acquire nosocomial infections, which are associated with ESBL-PE. For instance, UTI-related major RFs include previous use of antibiotics, invasive devices, indwelling urinary catheters, prior hospitalization, and a history of recurrent UTI (Castillo-Tokumori et al., 2017; Alqasim et al., 2018; Al-Jamei et al., 2019; Goyal et al., 2019). From bloodstream infection of older patients, the *bla*_TEM-1_ gene was the most prevalent, followed by *bla*_CTX-M-14_, *bla*_CTX-M-27_, and *bla*_CTX-M-65_ genes located on the incompatible plasmid group, which includes FrepB, FIA, FIC, K types (Xiao et al., 2019). The extensive and uncontrolled usage of *β*-lactam antibiotics led to the emergence of drug resistance, because of this, the selection of antibiotics should consider antimicrobial activity, pharmacokinetics, and information on causal agents (Hashemizadeh et al., 2019).

It is important to underline the role of IS*Ecp1* in the dissemination of ARGs. Recently, using upstream genetic structures of IS*Ecp1*-*bla*_CTX-M_, plasmids have been shown to contain *bla*_CTX-M_ transposition units. This information is useful to understand the location of IS*Ecp1* and classification of bacterial isolates harboring *bla*_CTX-M_ (Yagi et al., 2021). A clinical bone biopsy revealed a chromosomal and IncM2-type plasmid harbored *bla*_CTX-M-236_ gene, and the chromosomally located *bla*_CTX-M-3_ gene was found with IS*Ecp1* upstream and *orf*477 downstream in all copies of *Enterobacter hormaechei* (Figure 2A) (Huang et al., 2021). In blood samples of inpatients, the *bla*_CTX-M-15_ gene exists downstream of IS*Ecp1* while *bla*_KPC-3_ was between IS*Kpn7* and IS*Kpn6*; this was also the case with other transposons such as Tn*4401a*, *tnpR*, and *tnpA* in *K. pneumoniae* (Piccirilli et al., 2020). In infant blood, *bla*_CTX-M-14_, and *bla*_CTX-M-64_ were carried by IS*Ecp1*-mediated transposons Tn*6503a* and Tn*6502* (Yang et al., 2023). Likewise, in sputum samples, the *bla*_CTX-M-24_ gene occurred with an IS*Ecp1*-type element and carried IncFII plasmid (Yang et al., 2020). Further, a patient’s wound and fecal isolates in Switzerland was shown to exhibit *K. pneumoniae* ST23 and *E. coli* ST1 with the IncFII plasmid harboring *bla*_CTX-M-14_, which was inserted between IS*Ecp1* upstream and truncated IS*903B* downstream (Xavier et al., 2019). Transposition of IS*Ecp1* and other MGEs such as Tn*2*, Tn*3*, and IS*26* were observed with *bla*_CTX-M-15_ in the IncF- and IncI1-type plasmids (Zong et al., 2015), and the acquisition of these plasmids can make treatment options for resistant infections more difficult (Xavier et al., 2019). The plasmid- and IS*Ecp1*-mediated transmission of *bla*_CTX-M_ genes plays a significant role in the continued dissemination of ESBL resistance among *E. coli* populations that have the capacity to enter hospitals. Other RFs include hosts and sites of infection, such as infants infected with ESBL-PE (Marando et al., 2018) and the rectovaginal colonization of maternal ESBL-PE (Neemann et al., 2020). The ESBL-PE clones exist on newborns and on the hands of HCWs, which indicates a possible route of transmission among them (Dashti et al., 2010), as well as cross-contamination of environmental ESBL-PE (Boyer et al., 2015). The risk of ESBL-PE is higher in patients colonized with *K. pneumoniae* as compared to *E. coli*, and the presence of underlying diseases, such as malignant tumors, congestive heart failure, severe liver diseases, and peptic ulcers, can increase the risk further (Denkel et al., 2020). The risk of acquiring ESBL-producing *E. coli* (ESBL-EC) is higher in patients of an advanced age and with co-morbid medical conditions. Women are more at risk than men, and urban residents are at higher risk than rural residents (Laupland et al., 2008). Person-to-person communication (Ewers et al., 2012), mobile phones of HCWs (Loyola et al., 2016), ESBL-PE nasal colonization with hands (Founou et al., 2019), and feces in health care settings (NGuyen T. T. H. et al., 2019) are vital channels of AMR distribution.

## 4 Plasmid-mediated ESBLs in animal farms, FWs, and environmental settings

The distribution of antibiotic-resistant bacteria (ARB) changes and increases outside clinical settings. The prevalence of ESBL genes and plasmids in livestock, FWs, and farm environments are summarized in Table 2. Food animals are an essential part of our diet because they cover our protein requirements. From food animals and farms, ESBL-PE have been well documented in several countries, such as the United Kingdom, Germany, the Netherlands, Lebanon, Malaysia, and Thailand (Randall et al., 2011; Huijbers et al., 2014; Fischer et al., 2014; Gonggrijp et al., 2016; Tansawai et al., 2019; Seenama et al., 2019; Mobasseri et al., 2019; Velasova et al., 2019). Studies in China have been conducted on poultry (Liao et al., 2013), waterfowl (Yang et al., 2019; Zhang et al., 2023a; Zhang et al., 2024), pigs (Tan et al., 2023), and free-range Tibetan yaks, where ESBL genes were significantly associated with other ARGs (Rehman et al., 2017; Lei et al., 2018). In the USA and Spain, environmental flies in dairy, beef, and poultry farms have been shown to harbor ESBL genes (Solà-Ginés et al., 2015; Poudel et al., 2019). Close contact with livestock and introduction of new animals to the farm can impact the transmission and prevalence of ESBL-PE with co-localization of ARGs (Velasova et al., 2019). In addition, animals in the low- and middle-income countries are reared in poor bio-containment circumstances and with limited or no control over the use of antimicrobials (Carrique-Mas et al., 2015). Therefore, the poor biosecurity and farm environmental contamination can be strong reasons for ESBL-PE colonization in poultry, cattle, and pig farms (Hiroi et al., 2012; Dandachi et al., 2018; Gay et al., 2018).

**Table 2:** Selected studies from animal farms and human and environmental settings reporting ESBL-PE in various countries.

Category	Country	Bacteria isolated	Study on	Samples type	Total samples	Plasmid types	History/ techniques used	Identified genes/genotype/ ESBLs	Years of sampling	Reference
Animals and human contact	China	*Salmonella*	Human, Animadls	Human, Animadls	5,457	NA	WGS, cgMLST, cgSNP	*bla*_CTX-M-14_, *bla*_CTX-M-55_, *bla*_CTX-M-27_	2019–2020	Wang et al. (2023)
	Estonia	*E. coli*	Animal farmworkers	Nasal swabs, Stool	207	NA	ESBL-PE colonization in humans worked in contact with animals. A questionnaire, WGS, MLST	*bla*_CTX-M-1_, *bla*_CTX-M-1**5**,_ *bla*_CTX-M-14_	2012–2013	Telling et al. (2020)
	Nigeria	*E. coli*	Humans, Food Animals	Feces	454	FIA, FIB, FIC, FIIS	Monitoring of ESBL-PE in food animals and community. MIC, RBRT, PCR	*bla*_CTX-M_, *bla*_TEM_, *bla*_OXA_	2019	Adenipekun et al. (2019)
	Vietnam	*E. coli*	Non-intensive chicken farming	Feces	714	IncFIA, IncFII, IncFI, ColBS512, IncI1, IncHI2A	To investigate ESBL-EC colonization in human via non-intensive chicken farming. Questionnaire, MLST, WGS	*bla*_CTX-M-27_, *bla*_CTX-M-55_, *bla*_CTX-M-105_, *bla*_CTX-M-15_, *bla*_CTX-M-14_, *bla*_CTX-M-24_, *bla*_CTX-M-65_, *bla*_TEM-57_, *bla*_TEM-220_, *bla*_TEM-219_, *bla*_TEM-215_, *bla*_TEM-57_, *bla*_TEM-1_, *bla*_TEM-57_	2012–2013	Nguyen V. T. et al. (2019)
	Malaysia	*K. pneumonia*	Swine farms	Swine and human rectal swabs, Urine, Nasal swabs, Environment	389	NA	Resistant *K. pneumoniae* prevalence in healthy and unhealthy pigs, farmworkers, and farm environment. DDST, PFGE, PCR, conjugation	*bla*_CTX-M-1_, *bla*_CTX-M-2_, *bla*_CTX-M-15_, *bla*_TEM-1_, *bla*_SHV-11_, *bla*_SHV-12_, *bla*_SHV-61_	2013–2015	Mobasseri et al. (2019)
	Nicaragua	*E. coli* *K. pneumoniae*	Poultry, Wild birds, Human	Feces	300	NA	The relationship of ESBL-PE in humans, animals, and wild birds. PCR, ERIC2-PCR, MLST	*bla*_CTX-M-2_, *bla*_CTX-M-15_, *bla*_CTX-M-22_, *bla*_CTX-M-3_, *bla*_CTX-M-32_	2012	Hasan et al. (2016)
	Cameroon	*E. coli*	pig slaughterhouse	cotton swab, Stool	435	NA	slaughtered pigs and slaughterhouse workers. Disk diffusion, PCR, ERIC	*bla*_CTX-M,_ *bla*_TEM,_ *bla*_SHV_	2023	Matakone et al. (2024)
	Netherlands	*E. coli*	Horses	Feces	362	IncQ1, IncHI1, Col-like plasmids, IncF, IncY, IncI1, IncN	This study focuses on ESBL carriage in the open horse population and investigated the molecular characteristics, geographical distribution throughout the Netherlands. Sequencing, PCR, MLST, Questionnaires.	*bla*_CTX-M-2_, *bla*_CTX-M-14_, *bla*_CTX-M-15_, *bla*_CTX-M-32_, *bla*_SHV-12_, *bla*_CMY-2_, *bla*_ACT-10_	2020	Hordijk et al. (2020)
	Netherlands Belgium	*E. coli*	Broiler, Pig Farm.	Feces	1,596	NA	This study aimed to quantify ESBL-producing (ESBL-*E. coli*), carbapenem- and ciprofloxacin-resistant (CiproR) *Escherichia coli* in animal feces on broiler and pig farms with a history of high antibiotic use in Belgium and the Netherlands. MIC.	NA	2021	De Koster et al. (2021)
Animal farms	Brazil	*E. coli*	Cattle farms	Feces	191	IncI1	Intestinal colonization of ESBL-PE in healthy cattle. PFGE, MLST, DDST	*bla*_CTX-M-8_, *bla*_CTX-M-15_, *bla*_CTX-M-2_, *bla*_TEM-1_, *bla*_TEM-135_, *bla*_SHV-2a_	2014	Palmeira et al. (2020)
	Philippines	*E. coli*	Broiler farms	Cloacal and boot swabs,	156	NA	Prevalence study for ESBL-EC in broiler farms. DDST, PCR	*bla*_CTX-M-1_, *bla*_CTX-M-2_, *bla*_CTX-M-9_, *bla*_CTX-M-8_, *bla*_TEM_, *bla*_SHV_	2017	Gundran et al. (2019)
	Indonesia	*E. coli*	poultry-fish farms	chicken cloaca swabs, fish skin swabs, pond water, farmer hand swabs	256	NA	detect extended-spectrum ESBL-producing *E. coli* genes in integrated poultry-fish farms. DDST, PCR	*bla*_CTX-M_*, bla*_TEM_, *bla*_SHV_, *bla*_OXA-48_	2023–2024	Handayani et al. (2024)
	Germany	*E. coli*	Diseased food-producing animals	Feces	6,849	NA	GERM-Vet survey. DDST, Multiplex PCR, MIC, Phylo-grouping	*bla*_CTX-M-1_, *bla*_CTX-M-15_, *bla*_CTX-M-14_, *bla*_TEM-52_, *bla*_SHV-12_, *bla*_CTX-M-3_, *bla*_CTX-M-2_	2008–2014	Michael et al. (2017)
	South Korea	*E. coli*	Poultry and pig farms	Feces	281	IncFrep, IncFIB, IncI1, IncN	DDST, PCR, PBRT, PFGE, Conjugation	*bla*_CTX-M-65_, *bla*_CTX-M-14_, *bla*_TEM-1_, *bla*_CTX-M-15_	2009–2015	Shin et al. (2017)
	Tunisia	*E. coli*	Industrial poultry	Feces	137	IncI1, IncF, IncFIB, IncFIA, IncK, IncY, IncP, IncN	To study the transmission of ESBL-PE in the food chain. DDST, PFGE, MLST, PFGE	*bla*_CTX-M-1_, *bla*_CTX-M-15_, *bla*_CTX-M-14_, *bla*_TEM-1_	2013	Maamar et al. (2016)
Farm environment	Egypt	*E. coli* *K. pneumoniae*	Poultry house environment	Dust	28	NA	Farm dust was analyzed in laying hen farms. MIC, DDST	NA	2016–2017	Ahmed et al. (2020)
	USA	*E. coli* *K. pneumoniae*	Flies in poultry, livestock units, and the environment	Flies	493	NA	To identify the potential role of flies in the environmental spread of ESBL-PE. DDST, qPCR, WGS	*bla*_CTX-M-1_	2017	Poudel et al. (2019)
	India	*E. coli*	Livestock and poultry farm environment	Feces	78	B/O, FIC, A/C, Y, I1, HI1, N, L/M, HI2	Cattle, goat, pig, and poultry farms were analyzed. MIC, Multiplex PCR, MLST, PBRT	*bla*_CTX-M-1_, *bla*_CTX-M- 4_, *bla*_TEM_	NA	Tewari et al. (2019)
	Spain	*E. coli*	Poultry farm flies	Flies homogenate	682	IncI1, IncFIB	The spread of ESBLs in poultry farms surrounding environment was studied. MIC, PFGE, MLST, Phylo-grouping	*bla*_CTX-M-1_, *bla*_CTX-M-14_, *bla*_CTX-M-9_	2012	Solà-Ginés et al. (2015)
	USA	*E. coli*	Poultry house environment	Fecal, litter/compost, soil, swabs of feeders, waterers	780	IncFIB, IncI, IncFIC, IncFII, IncHIB, IncX, IncN, IncP, RepA, IncB/O/K/Z	DDST, MIC, WGS	*bla*_CTX-M-1_, *bla*_CTX-M-15_, *bla*_CTX-M-55_, *bla*_CTX-M-65_,	2021–2023	Parzygnat et al. (2024)
Copanion animals	Germany	*E. coli*	Experimental pig fattening facility	Faeces, Flies, dust	132	IncI1-I	Data were collected from samples of pig faeces, flies and dust, and piglets received specific medical food and medication on arrival at the farm. MALDI Biotyper Smart System GP, PCR, MIC, WGS	*bla*_CTX-M-1_	2019–2020	Behrens et al. (2023)
	China	*E. coli*	veterinary clinic	swab samples	98	NA	Investigated the ESBLs resistance genes of *E. coli* from companion animals. MALDI-TOF MS, DDST, Disk diffusion, PCR	*bla*_CTX-M_, *bla*_SHV_, *bla*_TEM_, *bla*_OXA_	2021	Cui et al. (2022)
	Japan	*E. coli*	animal shelter	Fecal samples or rectal swabs	333	NA	Cats and dogs in animal shelters. DDST, PCR, Sequencing,PFGE	*bla*_CTX-M-14_, *bla*_CTX-M-1_, *bla*_CTX-M-2_, *bla*_CTX-M-8_, *bla*_CTX-M-15_, *bla*_CTX-M-24_, *bla*_CTX-M-27_, *bla*_CTX-M-55_	2019	Umeda et al. (2019)
	Portugal	*E. coli*	companion animals and humans	Feces	229	NA	faecal samples were collected from healthy dogs, cats and their cohabiting humans. REP-PCR, WGS,	*bla*_CTX-M-15_, *bla*_CTX-M-55_	2018–2020	Menezes et al. (2023)
	Germany	*E. coli*	veterinary clinic	Fecal swabs	1,000	NA	Healthy and sick dogs. MALDI-TOF MS, Vitek2 Compact System	*bla*_CTX-M-14_	2016–2017	Werhahn Beining et al. (2023)
	Dutch	*Enterobacteriaceae*	dogs and cats and co-carriage with humans belonging to the same household	fecal sample	1,390	IncF, IncX4, IncI1, IncHI1, col-like, IncQ1	dogs and cats and co-carriage with humans belonging to the same household faecal sample. PCR, WGS	*bla*_CTX-M-1_*, bla*_CTX-M-15_*, bla*_CTX-M-32_*, bla*_SHV-12_*,bla*_TEM-52_	2014–2016	Van den Bunt et al. (2020)
	Brazil	*Enterobacteriaceae*	public center for zoonosis control (for dogs) and in a private shelter (for cats) and Veterinary Hospital	oral swab samples and urine samples	226	IncF, IncFII, IncI1, IncHI2, non-typeable plasmids	healthy stray cats and dogs and sick pets. API20E® strips and MALDI-TOF, disc diffusion, PCR, PFGE, PBRT	*bla_CTX-M-15_, bla*_CTX-M-2_, *bla*_CTX-M-9_, *bla*_CTX-M-8_	2012	Melo et al. (2018)
	New Zealand	*E. coli*	people and pets from the same household	urine and fecal samples	131	NA	Humans, cats and dogs. MALDI-TOF, disk diffusion, WGS	*bla*_CTX-M-15_*, bla*_CTX-M-27,_ *bla*_CTX-M-14_	2015–2017	Toombs-Ruane et al. (2020)
	South Korea	*E. coli*	veterinary hospitals	diarrhea, skin, ear canals, urine, and genitalia	836	NA	the diarrhea, skin, ear canals, urine, and genitalia of dogs and cats. MALDI-TOF, MIC, PCR, sequences, MLST, PFGE	*bla*_CTX-M-3_*, bla*_CTX-M-14_*, bla*_CTX-M-15_*, bla*_CTX-M-55_*, bla*_CTX-M-65_	2018–2019	Choi et al. (2023)
	Japan	*E. coli*	Veterinary Medical Center	Fecal samples	678	IncFIB, IncI2	DDST, MIC, WGS, Conjugal transfer of plasmids, PCR	*bla*_CTX-M-1_, *bla*_TEM_, *bla*_SHV_	2018–2022	Yasugi et al. (2023)

MLST, Multi-locus sequence typing; pMLST, plasmid Multi-locus sequence typing; PFGE, Pulsed-field gel electrophoresis; PCR, Polymerase chain reaction; MIC, Minimum inhibitory concentration; DDST, Double disc synergy test; WGS, Whole-genome sequencing; GERM-Vet survey, German national monitoring program; PBRT, PCR-based replicon typing; NA, Not analyzed/available.

IS*Ecp1* is often present upstream of the *bla*_CTX-M_ genes while IS*903* is in the downstream region in several replicon types, such as IncF, IncI1-Iγ, IncFIB, IncN, and IncP, which play a vital role in the capture, mobilization, and expression of *bla*_CTX-M_ genes (Tamang et al., 2013; Ben Said et al., 2016). For instance, *Salmonella enterica serovar Virchow* isolated from carcasses and fecal samples of chickens (*n* = 256), pigs (*n* = 7), and cattle (*n* = 2) in South Korea exhibited the IncHI2 conjugative plasmid harboring the *bla*_CTX-M-15_ gene with transposition of upstream IS*Ecp1* and downstream orf477 (Na et al., 2020). Although the isolate rate is low, *E. coli* ST405 contains IncFII-type plasmid harboring *bla*_CTX-M-14_ gene with transposition of upstream IS*Ecp1* and downstream truncated IS*903*, which was isolated from pasture-based dairy farms in New Zealand (Burgess et al., 2021).

Specifically, from the perspective of poultry, air-borne dust affects the health status of laying hens (Gole et al., 2014). Meanwhile, ESBL-PE were recorded in the air-deposited dust on eggs in Egyptian hen coops (Ahmed et al., 2020). A low level of vertical transmission of ESBL genes has been reported from hatchery chicks, with a history of ESBL genes in parent poultry flocks (Projahn et al., 2017). Backyard poultry are involved in the dissemination of ESBL genes in poultry farms, FWs, and environmental settings by IncF-, IncY-, IncX-, IncHI1-, IncFIA-, and IncI1-type plasmids (Tansawai et al., 2019). Due to direct contact with broiler chickens, a higher level of ESBL-EC prevalence was recorded among the farm-based individuals compared to community living around organic Dutch broiler farms (Huijbers et al., 2015). Identical genes, such as *bla*_CTX-M-65_, and *bla*_CTX-M-55_, have been observed in non-intensive chicken farms and human beings. By whole-genome sequencing (WGS), 29 plasmid incompatibility groups of those 486 ESBL-EC isolates were noted, with IncFIA, IncFI, IncFII and ColBS512 predominant in human isolates, while Col156, IncQ1, and IncHI2A were predominant in chicken isolates (Nguyen V. T. et al., 2019). A recent study on a poultry farm showed the *bla*_CTX-M-1_ gene in IncI1/ST3 plasmids and/or chromosomes, which harbored an IS*Ecp1* element in the upstream region of this gene (Aldea et al., 2022). Thus, the plasmid-mediated transmission of ESBL genes plays an important role in AMR spread in the poultry production system and related environmental settings (Projahn et al., 2017; Oikarainen et al., 2019).

Plasmid-mediated ESBL genes transmission has been reported in pigs and pig FWs in several countries such as Denmark, Germany, Switzerland, and the Netherlands (Hammerum et al., 2014; Dohmen et al., 2015; Dahms et al., 2015; Kraemer et al., 2017). The fecal ARG load is higher in pig FWs compared to broiler FWs and livestock. For instance, *bla*_CTX-M-1_, *bla*_CTX-M-14,_ and *bla*_SHV-12_ were observed in both humans and pigs on the same farms, while *bla*_CTX-M-1_ was detected on IncI1-, IncF-, or IncN-type plasmids (Hammerum et al., 2014). In a recent study, high clonal correlation of ESBL-EC strains was also observed in pig and human isolates from slaughterhouses (Matakone et al., 2024). The CTX-M-1 group is present in the piggery, air, and dust, which highlights the possibility of transmission between animals and humans after direct contact (Dohmen et al., 2017). Human and porcine isolates contained identical *bla*_CTX-M-1_ genes in *E. coli* ST453 with IncI1-type plasmid, showing the potential for clonal transmission (Dohmen et al., 2015). The rectal carriage of ESBL-PE occurred in pig-FWs, as in the general population of Germany. Isolation of clonally identical ST10/CTX-M-1 in FWs and the farm environment suggested the possibility of cross-transmission (Fischer et al., 2017). A study in Brandenburg, Germany, found that ESBL-EC isolated from flies showed genetic similarities to swine fecal isolates, indicating the fly-associated transport of diverse ESBL-EC from the pig farm into urban habitation areas (Behrens et al., 2023). Therefore, pigs and farmers can contribute to the spread of ESBL-PE, which can lead to the spillover of ARB to the community.

## 5 The prevalence of ESBL genes in the food chain

It is important to monitor the distribution of ESBL-PE through the food chain. Reports have shown the occurrences of ESBL-PE in poultry, swine, bovine, and fishes (Table 3). However, sequence data shows that ESBL-EC and *K. pneumoniae* isolates from human-polluted environments are genetically dissimilar from those polluting food items (Martak et al., 2022). The ESBL-PE with the highest prevalence of *bla*_TEM_, followed by *bla*_CTX-M_ and *bla*_SHV_, was found in a variety of retail foods including frozen chicken, raw chicken meat, and pork. (Ye et al., 2018). For instance, 11 STs of *E. coli* carrying *bla*_CTX-M-1_, *bla*_CTX-M-14_, *bla*_CTX-M-15_, and *bla*_TEM-1_ genes were identified in fresh pork meat in Germany (Schill et al., 2017). The identification of *E. coli* isolates carrying *bla*_CTX-M-8_, *bla*_CTX-M-14_, *bla*_CTX-M-15_, and *bla*_CTX-M-55_ in retail meat products from supermarkets across the United Arab Emirates highlights a significant prevalence of these ARBs (Habib et al., 2023). Notably, one-day old chicks imported from Brazil to Uruguay were shown to act as the “Trojan horse,” allowing the latent spread of *E. coli* carrying *mcr*-9, *bla*_CTX-M-2_, *bla*_CTX-M-8_, *bla*_CTX-M-15_, *bla*_CTX-M-55_, and *bla*_CMY-2_ in poultry farms (Coppola et al., 2022). The *E. coli* ST48 isolated from camels was harboring *bla*_CTX-M-15_ in Kenya, which can threaten public health through direct contact or via the food chain (Nüesch-Inderbinen et al., 2020).

**Table 3:** Selected studies from food chain and wildlife environmental settings reporting ESBL-PE in various countries.

Category	Country	Bacteria isolated	Study on	Samples Type	Total samples	Plasmid types	History/ techniques used	Identified genes/genotype/ESBLs	Years of sampling	Reference
Food chain	Tunisia	*E. coli* *K. pneumoniae*	Wild fish	Gills, Stomach contents	126	NA	Prevalence of ESBL-PE in intestine and gills of wild fish. DDST, PCR	*bla*_CTX-M-1_, *bla*_CTX-M-15_, *bla*_TEM-1-a_	2016	Hassen et al. (2020)
	Egypt	*E. coli* *K. pneumoniae* *Enterobacter* spp.	Retail chicken meat	Chicken carcasses	106	NA	Prevalence of ESBL-PE in the food chain of Egypt. DDST, PCR, Sequencing	*bla*_CTX-M-15_, *bla*_CTX-M-14_, *bla*_SHV_	2013	Abdallah et al. (2015)
	Senegal	*E. coli*	Open wet markets	Broilers	240	NA	Aimed to assess the prevalence, resistance profile, and carriage of ESBL-encoding genes in ESBL-Ec isolates from broilers in two markets. MALDI-TOF, DDST, Disk diffusion, PCR,	*bla*_CTX-M-1-group_, *bla*_CTX-M-2-group_, *bla*_CTX-M-8-group_, *bla*_CTX-M-9-group_, *bla*_TEM,_	2018–2019	Cissé et al. (2024)
	China	*E. coli*	Poultry farm and market	Feces, Meat, Liver	195	IncN, IncFIB, IncI1, IncHI2, IncHI1	To study the prevalence of ESBLs in chicken farms and live chicken markets. MIC, DDST, PFGE, PCR, Conjugation	*bla*_TEM-1_, *bla*_SHV-5_, *bla*_CTX-M-15_, *bla*_CTX-M-65_, *bla*_CTX-M-55_, *bla*_CTX-M-14_, *bla*_CTX-M-3_, *bla*_CTX-M-13_, *bla*_CTX-M-9_, *bla*_CTX-M-79_, *bla*_CTX-M-101_, *bla*_CTX-M-1_, *bla*_CTX-M-64_, *bla*_CTX-M-123_, *bla*_CTX-M-132_	2011–2013	Tong et al. (2015)
	Portugal	*E. coli*	Poultry Pigs Rabbits	Feces, Macerated organs	179	IncI1	Animals are involved in the spread of ESBLs in the food chain. DDST, PCR, MLST	*bla*_CTX-M-1_, *bla*_CTX-M-14_, *bla*_SHV-12_	NA	Jones-Dias et al. (2016)
	France	*E. coli*	Retail chicken meat	Meat	97	IncI1, IncX1, IncA/C	To study the effects of reduced usage of antibiotics in poultry production. DDST, simplex PCR, PFGE, PBRT	*bla*_CTX-M-1_, *bla*_TEM-52_, *bla*_SHV-12_	2015–2016	Casella et al. (2017)
	Pakistan	*E. coli*	Food chain	Feces, Eggs, Mastitis milk	200 200 750	NA	ESBL-PE prevalence in food animals in Pakistan and China DDST, PCR	*bla*_CTX-M-15_, *bla*_CTX-M-14_, *bla*_CTX-M-3_, *bla*_CTX-M-1_, *bla*_CTX-M-55_, *bla*_TEM-1_, *bla*_SHV-1_, *bla*_SHV-12_	2015–2016	Rahman et al. (2018)
	South Korea	*Salmonella*	Retail Meats	Beef, Pork, Chicken, Duck samples	1876	IncFIB, IncFII, IncQ1, IncHI2, IncHI2A	Vitek MS, MIC, WGS	*bla*_CTX-M-15_	2018–2019	Kang et al. (2024)
	Brazil	*E. coli* *K. pneumoniae*	Retail chicken meat	Carcasses	105	NA	Seven market places in Brazil were investigated. MIC, DDST, PCR	*bla*_CTX-M-1_, *bla*_CTX-M-2_, *bla*_CTX-M-8_	2011–2013	Casella et al. (2015)
Wildlife	North America	*E. coli*	Crow	Droppings, Water	282	NA	To examine the threat of wild birds as a vector of AMR to the environment. MLST, DDST, PCR	*bla*_CTX-M_, *bla*_TEM_, *bla*_SHV_	2014–2015	Sen et al. (2019)
	Brazil	*E. coli* *K. pneumoniae*	wild birds	Feces	80	NA	MALDI-TOF MS, DDST, disk diffusion, PFGE, PCR	*bla*_CTX-M-group_, *bla*_TEM_, *bla*_SHV_	2021	Ramos et al. (2024)
	USA	*E. coli*	Cattle, Coyotes, Feral swine,	Feces, soil, water	477	IncR, IncN, IncFIB, IncFIA, Col440I	PCR, WGS, disk diffusion, Shotgun metagenomic sequencing,	*bla*_CTX-M-1_, *bla*_CTX-M-32_	2017–2018	Liu et al. (2024)
	Spain	*E. coli*	Eurasian griffon vultures	cloacal swabs	218	NA	ESBL-PE isolated from Eurasian Griffon (*Gyps fulvus*). PCR, MIC, WGS, MLST	*bla*_CTX-M-1_, *bla*_CTX-M-14_, *bla*_CTX-M-15,_ *bla*_CTX-M-27_, *bla*_CTX-M-55_, *bla*_CTX-M-65_	2019–2020	Guitart-Matas et al. (2024)
	China	*E. coli*	Wild birds	Faecal and swab samples	4,422	IncFIB, IncFIC, IncFII, IncY	ESBL-pe separation from highland migratory birds. 16S rDNA, BD Phoenix, MIC, Conjugation, PCR, WGS	*bla_CTX-M-1_, bla*_CTX-M-14_*, bla*_CTX-M-15_*, bla*_CTX-M-27_*, bla*_CTX-M-55_	2018–2013	Wang et al. (2024)

MLST, Multi-locus sequence typing; pMLST, plasmid Multi-locus sequence typing; PFGE, Pulsed-field gel electrophoresis; PCR, Polymerase chain reaction; MIC, Minimum inhibitory concentration; DDST, Double disc synergy test; WGS, Whole-genome sequencing; PBRT, PCR-based replicon typing; NA, Not analyzed/available.

Since poultry is a common and affordable source of protein, the screening of industrial poultry production systems for ESBL-PE is essential (Maamar et al., 2016). For example, Finland stopped using antimicrobials in the broiler production system in 2009 (Päivärinta et al., 2016). Although at lower rates than other European countries, the various flocks of broiler chickens without antimicrobial usage also revealed seven ESBL-EC STs in Finland, harboring a range of plasmid incompatibility groups, such as IncB/O/K/Z, IncI1, IncFII, IncFIB, IncII, IncX1, and IncFIC (Päivärinta et al., 2020). Brazil is the third largest chicken meat producer and also has a huge local consumer market (Alves et al., 2012; Palmeira et al., 2020). In Brazil, the chicken meat acts as a reservoir of *bla*_CTX-M-2_, harboring a complex-class 1 integron, with IS*CR1*-linked sequence to *dfr* and/or *aadA* gene cassettes, which could be a community health hazard (Casella et al., 2015).

Beef and veal are another important food protein source. Worryingly, 68.2% of tested beef farms in Brazil were positive for ESBL-EC, with a high prevalence of *bla*_CTX-M-8_ carried by IncI1/pST113-type plasmids linked to ST648 and ST155 clones (Palmeira et al., 2020). Surprisingly, environmental contamination has been verified from sludge cultures on Dutch dairy farms, with seven different ESBL genes (Gonggrijp et al., 2016). High numbers of ESBL genes such as *bla*_TEM-1_, which poses a direct threat to the health of dairy cows and further affects the quality of dairy products, were observed in the bedding material (Yang et al., 2021), Finally, the presence of IS*Ecp1* with *bla*_CTX-M_ in Indian dairy isolates highlights the potential role of the IS*Ecp1*-like element in the spread of this particular gene into the food chain (Koovapra et al., 2016).

## 6 Contribution of international traveling and import of animal products to the spread of ESBL-PE

There are multifactorial reasons for the rapid spread of ESBL-PE, such as migration and human travel (Arcilla et al., 2017; Doi et al., 2017) and imported animal meat products (Kim et al., 2018). However, travel restrictions alone may not influence or limit the spread of ESBL genes (Howard-Jones et al., 2022). We have limited knowledge on the colonization of ESBL-PE through travel among different countries concerning humans and animals. For instance, imported meat in South Korea revealed a high prevalence of *E. coli* in poultry as well as pork and beef. *bla*_CTX-M-94_ has been reported in Brazilian and US poultry meats, *bla*_CTX-M-58_ was reported in pork meat in France, *bla*_CTX-M-79_, *bla*_CTX-M-1_ in Hungary, *bla*_CTX-M-14/18_ in Spain, *bla*_CTX-M-1_ and *bla*_CTX-M-2_ in Chile on multiple plasmid incompatibility groups, such as FIA, FIB, FIC, Frep, FIIA, HI1, and HI2 (Kim et al., 2018). In Ghana, *E. coli* ST10, ST38, and ST155 clones carrying *bla*_CTX-M-15_ and *bla*_CTX-M-1_ and *K. pneumoniae* ST2570, ST147, and ST15 clones carrying *bla*_CTX-M-15_ and *bla*_CTX-M-14_ have been identified in imported poultry meat from Belgium, Germany, Brazil, the USA, Poland, and the Netherlands (Eibach et al., 2018). Legal and illegal poultry/pig meat and matchmaking products imported into Germany from countries such as Brazil, Chile, and Egypt have shown evidence of *E. coli* ST101, ST117, ST7509, ST7845, and ST7602 clones primarily containing *bla*_CTX-M-2_ along with *bla*_CTX-M-8_, *bla*_CTX-M-1_, and *bla*_CTX-M-9_ (Müller et al., 2018). In sub-Saharan Africa, up to 54% of poultry meat from Europe and Brazil is regularly contaminated with ESBL-EC (Olaru et al., 2023). Further, from imported food products in the USA, *S. enterica* was isolated in frozen octopus carrying *bla*_TEM-1_-positve plasmid with IncN type, whereas *bla*_TEM-1_ and *bla*_CTX-M-9_ were harbored on IncI1 plasmid in frozen tilapia fish (Bae et al., 2015).

Frequent trips between countries increases the spread of ESBL-PE (Table 4). International travel, the presence of gastrointestinal comorbidity, and prior UTIs are important in the acquisition of ESBL-PE (Strysko et al., 2016; Woerther et al., 2017; Wuerz et al., 2020). Travelers who visit relatives and friends are at lower risk of acquiring ESBL-PE (Schaumburg et al., 2019). Moreover, antibiotic therapy during travel (Angelin et al., 2015; Wuerz et al., 2020), travel diarrhea (López-Vélez et al., 2022), UTI during travel (Patjas and Kantele, 2022), and type of accommodation (Meurs et al., 2019) are important factors in ESBL-PE colonization. Reports confirmed colonization of *bla*_CTX-M-15_, *bla*_CTX-M-3_, *bla*_CTX-M-55_, *bla*_CTX-M-14_, and *bla*_CTX-M-27_ after traveling to Sub-Saharan Africa, North Africa, the Middle East, South Asia, South America, Central Asia, Central America, and Oceania from Japan (Mizuno et al., 2016; Nakayama et al., 2019). One study showed that 75% of AMR plasmids found in the feces of travelers with a history of travel to Laos contained ESBL genes (Snaith et al., 2023). Research showed German travelers to India received the highest acquisition of *bla*_CTX-M-15_, while travelers to South-East Asia were colonized with *bla*_CTX-M-27_, and Southern Africa and South America travelers acquired *bla*_CTX-M-15_ (Lübbert et al., 2015). Similarly, in another study from Germany, 21% of travelers to East Africa, 23% of travelers to Southeast Asia, and 42% to South Asia returned with ESBL-PE (Meurs et al., 2019). The UK’s findings supported the argument that travelers with a trip history to Asia or Africa carried *bla*_CTX-M_ (Bevan et al., 2021; Otter et al., 2019). In Dutch travelers, higher colonization of *bla*_CTX-M-15_ and *bla*_CTX-M-14/18_ was recorded before and after travel to Asia, Africa, and Oceania (Arcilla et al., 2019). Finnish travelers with a travel history outside the Nordic countries were found to be predominantly positive with *bla*_CTX-M-15_ followed by *bla*_CTX-M-1_, *bla*_CTX-M-9_, *bla*_TEM_, and *bla*_SHV_ (Kantele and Lääveri, 2021). The high rates of acquisition of the intestinal *bla*_CTX-M-15_, *bla*_CTX-M-1_, *bla*_CTX-M-14_, *bla*_CTX-M-27_, and *bla*_CTX-M-65_ carried by the IncF-type plasmids were observed in French soldiers who served in Afghanistan for 4–6 months without inter-individual transmission (Maataoui et al., 2019). Consequently, ongoing reporting and monitoring of ESBL-PE among travelers is required to mitigate the emerging challenge of AMR.

**Table 4:** Selected studies from travelers reporting ESBL-PE in various countries.

Country	Bacteria isolated	Study on	Sample types	No. of samples	History/ techniques used	Identified genes/genotypes/ ESBLs	Years of sampling	Reference
Germany	*E. coli* *K. pneumoniae*	Travelers to low and middle-income countries	Fecal	230	A prospective cohort study, to analyze the effect of traveling to high-risk areas on the spread of ESBL-PE. MIC, Questionnaires	NA	2016–2017	Meurs et al. (2019)
France	*E. coli*	Military travelers	Fecal	201	Prospective study, to analyze ESBL-PE accusation in French military personnel working overseas for 4–6 months, Questionnaire, rep-PCR, WGS	*bla*_CTX-M- 15_, *bla*_CTX-M- 27_, *bla*_CTX-M- 1_, *bla*_TEM- 1_	2012	Maataoui et al. (2019)
Finland	*E. coli* *K. pneumonia*	International travelers	Patients with positive urinary culture	430	Factors associated with ESBL-PE were explored in relation to (i) any UTI compared to controls, (ii) ESBL-PE UTI compared to controls, and (iii) ESBL-PE UTI in contrast to non-ESBL-PE UTI.	NA	2015–2020	Patjas et al. (2024)
Netherland	*E. coli*	Dutch travelers	Fecal	2,216	Cross-sectional study, to access ESBL-PE colonization in Dutch population on international travel. Questionnaire, MIC, DDST	*bla*_CTX-M- 15_, *bla*_CTX-M 14/18_, *bla*_CTX-M-9_, *bla*_CTX-M-1_, *bla*_CTX-M-27_, *bla*_CTX-M-55/57_, *bla*_CTX-M-3_, *bla*_CTX-M-32_, *bla*_CTX-M-65_, *bla*_CTX-M-24_, *bla*_CTX-M-38_, *bla*_SHV-12_, *bla*_SHV-2a_, *bla*_SHV-28_, *bla*_TEM-52c_, *bla*_TEM-176_, *bla*_VEB_	2012–2013	Arcilla et al. (2019)
France	*E. coli*	Military and civilian travelers	Feces	166	Cross-sectional study, to identify RFs. Questionnaire, DDT, DDST	NA	2012–2015	Flateau et al. (2018)
Japan	*Enterobacteriaceae*	Business travelers	Stool	192	ESBL-PE was studied in Japanese long-term business travelers. PCR, Sequencing	*bla*_CTX-M-15_, *bla*_CTX-M-3_, *bla*_CTX-M-55_, *bla*_CTX-M-14_, *bla*_CTX-M-27_	2012–2015	Mizuno et al. (2016)
Netherlands	*E. coli* *K. pneumoniae*	International travelers	Feces	2,216	A prospective multicenter cohort study in Dutch travelers and non-traveling household members. Questionnaire, MIC, DDST, PCR	*bla*_CTX-M-9_, *bla*_CTX-M-15_	2012–2013	Arcilla et al. (2017)
British	*E. coli* *K. pneumonia*	military personnel	Feces	113	ESBL-PE was studied to find out how common it is among British military personnel. PCR	*bla*_CTX-M_, *bla*_SHV_, *bla*_TEM_	2021–2022	Toriro et al. (2024)
The United States.	*E. coli* *K. pneumoniae*	Children with travel history	blood, Urine, Respiratory/ abdominal fluid/abscess or skin swabs	1,258	Case–case–control study in 0–18 years children in US hospital to identify RFs. VITEK2 machine	NA	2012–2014	Strysko et al. (2016)
Sweden	*E. coli*	healthcare scholars on medical assignments traveling abroad	Feces	99	Prospective study, to investigate RFs linked with ESBL-PE colonization in health care students. DDT, DDST, PFGE	NA	2010–2014	Angelin et al. (2015)
Germany	*E. coli* *K. pneumoniae*	Returning international travelers	Feces	225	Prospective cohort study, ESBL-PE colonization was studied in travelers to 53 countries. PCR, Sequencing	*bla*_CTX-M-15_, *bla*_CTX-M-27_, *bla*_CTX-M-14_, *bla*_CTX-M-65_, *bla*_CTX-M-55_, *bla*_SHV-12_	2013–2014	Lübbert et al. (2015)

PFGE, Pulsed-field gel electrophoresis; PCR, Polymerase chain reaction; MIC, Minimum inhibitory concentration; DDT, Disk diffusion test; DDST, Double disc synergy test; WGS, whole-genome sequencing; NA, Not analyzed/available.

## 7 The prevalence of ESBL genes in wastewater and agriculture settings

About 20 million hectares of arable land are irrigated with wastewater globally (Mateo-Sagasta et al., 2013); this wastewater comes from private use, slaughterhouses, animal farms, and hospital outflows that contain levels of ARGs (Dickin et al., 2016; Azuma et al., 2022). Thus, soils irrigated with untreated wastewater act as a reservoir for ARGs (Bougnom et al., 2020). For example, the occurrence of *bla*_CTX-M-3_, *bla*_CTX-M-12_, *bla*_CTX-M-14_, and *bla*_CTX-M-15_, as well as *bla*_TEM_ and *bla*_SHV_, on spinach farms was alarming where river water was used to irrigate land (Richter et al., 2020). Wastewater from dairy farms in agricultural fields implies the spread of the *bla* genes in environmental settings. The *bla*_TEM-1_ gene was recorded in dairy waste even after the treatment of solid manure and dairy sewage (Yang et al., 2021).

Water from estuaries, wells, and springs used for human activities, watering animals, and crop irrigation without treatment is a source of CTX-M-15-producing *E. coli* ST10, ST38, and ST131 clones and *Klebsiella* isolates ST323, ST17, and ST405, which proposes a complex origin of contamination (Diab et al., 2018; Hooban et al., 2022). In addition, freshwater environments are vulnerable to antibiotic pollution from a variety of sources, including sewage, agricultural runoff, and farm leakage, creating an ideal environment for the colonization, multiplication, and spread of ARB and facilitating their accumulation, evolution, and spread (Cho et al., 2023). The level of ARB is highest in the river systems, followed by lakes, dams, ponds, and spring water (Nnadozie and Odume, 2019). The aquatic environment that receives wastewater in the Kashmir Valley, India, has shown coexistence of *bla*_TEM_ and *bla*_CTX-M-15_ with MGEs such as IS*Ecp1*, Tn*21*, and Tn*3*, associated with plasmid incompatibility groups such as B/O, HI1, HI2, N, I1, FIB, and FIA (Sultan et al., 2020; Sultan et al., 2022).

WWTP includes primary and secondary procedures to remove organic matter, pollutants, and microbes. However, these practices have some limitations in eradicating fecal coliforms and ARGs (Li et al., 2016; Smyth et al., 2020). Municipal-treated wastewater can be a potential source of MDR *E. coli* and ARGs in surface waters (Kutilova et al., 2021). ESBL genes, such as *bla*_TEM_, *bla*_SHV-12_, *bla*_CTX-M-1,_ and *bla*_CTX-M-15_ carried by IncF-, IncI1-, IncHI1/2- and IncA/C-type plasmids, were identified in urban WWTP effluents (Smyth et al., 2020). The chloroform treatment process at WWTP reduces the number of *E. coli*, but there is still some ESBL-EC released into the environment through the effluent, the most prominent gene being *bla*_CTX-M-9_ (Xie et al., 2023). A wastewater treatment plant in Colombia receives domestic, hospital, and industrial wastewater and releases effluent into the natural water source. Samples of raw influent, recycled sludge, aeration tanks, and final effluents revealed diverse ESBL-EC clones with a high prevalence of *bla*_TEM_, followed by *bla*_CTX-M-1_, *bla*_CTX-M-9_, *bla*_CTX-M-8/25_, and *bla*_CTX-M-2_ (Aristizábal-Hoyos et al., 2019). UV disinfection is one of the practices used to eliminate ARB in WWTPs. UV treatment also reduced ARB in effluents (Silva I. et al., 2018). However, different genetic arrangements such as integrase gene, variants of *bla*_CTX-M_ genes like *bla*_CTX-M-15_, *bla*_CTX-M-32_, *bla*_CTX-M-1_, *bla*_CTX-M-27_, and *bla*_CTX-M-14_ flanked by IS*Ecp1*/IS*26*, IS*903,* and orf477, have been observed in UV-treated effluent (Silva I. et al., 2018). Weather can also affect the presence of ESBL-PE in WWTP effluents, as greater quantities were observed in colder months than in warmer months (Li et al., 2023). A range of *bla* genes were identified from wastewater, sewage sludge, and WWTP effluents including *bla*_VIM_, *bla*_GES_, *bla*_CTX-M-9_, and *bla*_CTX-M-1_ (Schages et al., 2020). Despite the treatment, WWTP effluents contained bacteria that could contaminate river water and air nearby grit tanks or bioreactors, primarily *bla*_CTX-M-3_, *bla*_CTX-M-9_, and *bla*_CTX-M-1_ followed by *bla*_TEM-49_, and *bla*_SHV-2_ genes (Korzeniewska and Harnisz, 2013b). Improved wastewater treatment is therefore needed to effectively monitor and control the spread of priority MDR pathogens and ESBL-PC in the population (Puljko et al., 2024).

Worrisomely, ARGs were abundant in untreated hospital wastewater, municipal WWTPs, and river sediments (Yuan et al., 2021). Untreated wastewater effluents from the sick bay contained *bla*_CTXM-1_, *bla*_CTXM-2_, *bla*_CTXM-9_, *bla*_CTXM-8_, *bla*_CTXM-25_, and *bla*_TEM_, which highlights the need for hospital-based wastewater treatment facilities (Adekanmbi et al., 2020). Moreover, a predominance of *bla*_TEM_, followed by *bla*_SHV_ and *bla*_CTX-M_ genes, have been noted from surface river water sites closer to human anthropogenic actions, such as hospital, industrial, and municipal wastes, from Ganga, India (Chaturvedi et al., 2020). Therefore, monitoring of AMR in WWTP effluents is necessary to understand the load of fecal coliforms, ARGs, MGEs, and ARB into the natural environment. This will strengthen our ability to decide on adding more treatment technologies to reduce the influx of ARGs to aquatic environments (Smyth et al., 2020).

## 8 The prevalence of ESBL genes in companion animals

Companion animals play a major role in the lives of many people and allow the transmission of ARB between people and animals (Dickson et al., 2019). Surprisingly, the *bla*_CTX-M-15_ gene was widespread in both humans and pets, with the possibility of gene exchange between them (Bogaerts et al., 2015). Occurrence of *bla*_CTX-M-15_ was highest in pets originating from shelters (Johansson et al., 2022), followed by livestock areas of county fairs, livestock auction markets, equine facilities, and dairy farms (Adams et al., 2018). Pets participate in the dissemination of the ESBL-EC. For instance, 13 distinct STs with the most common ST117, ST131, and ST38 clones containing eight variants of the *bla*_CTX-M_ gene including *bla*_CTX-M-14_ and *bla*_CTX-M-15_ have been identified in animal shelters (Umeda et al., 2019). A German study indicated that 8.9% of dogs seen in veterinary clinics carried ESBL-EC, and that seven factors, including husbandry system, contact with puppies, and feeding raw meat, were significantly associated with ESBL-EP colonization (Werhahn Beining et al., 2023). The most common transmission and occurrence of *bla*_CTX-M-1_, followed by *bla*_CTX-M-15_ and *bla*_CTX-M-32_ harboring IncI1- and IncF-type plasmids, have been documented in dogs and their owners (Van den Bunt et al., 2020). A similar study on stray dogs and cats confirmed them as a significant source of MDR and ESBL-PE containing *bla*_CTX-M-15_, *bla*_CTX-M-2_, *bla*_CTX-M-9,_ and *bla*_CTX-M-8_ with predominantly ST973, ST457, ST90, and ST2541 clones harboring different plasmid types (Melo et al., 2018). A pet like a dog can contribute to the spread and community transmission of ESBL-EC clonal types ST131, ST963, and ST69, as similar strains were isolated from family members and pets within the same household (Toombs-Ruane et al., 2020). In Korea, third-generation cephalosporin- and fluoroquinolone-resistant *E. coli* strains were widely distributed in cats and dogs, with *E. coli* ST131 harboring *bla*_CTX-M-14_ and *bla*_CTX-M-15_ genes and *E. coli* ST405 harboring the *bla*_CMY-2_ gene (Choi et al., 2023). Notably, ESBL-EC carrying silenced *mcr*-9 were isolated from companion cats in Japan, albeit not universally (Yasugi et al., 2023). Moreover, *E. coli* ST224 contains *bla*_CTX-M-8_ and identifies IncI1-, IncY-, IncFIA-, and IncFIB-type plasmids in domestic cats, which may create therapeutic limitations in veterinary medicine (Silva M. M. et al., 2018). Thus, pets are involved in the spread of ESBL-PE to other animals, the environment, and humans via saliva or direct contact (Melo et al., 2018). Therefore, strict monitoring of companion animals is required to limit the spread of ESBL-PE.

## 9 The prevalence of ESBL genes in wildlife

Rising populations lead to the destruction of natural wildlife habitats, which in turn unexpectedly increases the role of wildlife in ARG dissemination. This includes birds (Marcelino et al., 2019) and wild animals such as wild ungulates, wild boar (*Sus scrofa*), red deer (*Cervus elaphus*), fallow deer (*Dama dama*), and mouflon (*Ovis aries subsp. musimon*) (Torres et al., 2022). This is because animals need food reservoirs and water sources; thus, they travel from their native habitats to other places. This causes wildlife to come in to contact with anthropologically affected areas that may hold residual antibiotics and/or ARB (Carroll et al., 2015).

Wild birds are assumed as significant reservoirs and propagators of ARGs that can contaminate the environment (Dolejska and Literak, 2019). Migratory birds can further enhance ARG dissemination widely over long distances, which ultimately enhances worldwide occurrences of AMR (Hernando-Amado et al., 2019). For example, many reports have been documented for the existence of ARB in wild birds, included but not limited to geese and ducks (Li et al., 2024; Zhang et al., 2023b; Zhang et al., 2023c), gulls (Hernandez et al., 2013; Ruzauskas and Vaskeviciute, 2016), rooks (Oravcova et al., 2013), and passerines (Dolejská et al., 2008). Moreover, wild birds show higher ARGs in anthropologically stimulated environments than those existing in less inhabited zones (Miller et al., 2020; Marcelino et al., 2019). A recent study revealed the influence of microbial community structure, MGEs, and residual antibiotics on resistomes of bird feces and further found great interconnectivity of ARGs among the microbiomes of wild birds and their habitats (Luo et al., 2022).

## 10 ESBL-PE and HGT

Moreover, MGEs such as integrons, plasmids, and transposons are involved in the horizontal spread of ARGs in humans, animals, and the environment. ESBL-PE bacteria are increasingly found across various environments, with *bla*_CTX-M-15_ being widespread. MGEs, including plasmids and transposons, facilitate the spread of antibiotic resistance among bacteria (Zhang et al., 2020). MGEs play an important role in HGT and the association of IS*CR1* elements recorded with *bla*_CTX-M-15_ (Ali et al., 2016), and the same gene has been recognized by both community and cattle linked by MGEs such as Tn*3* or IS*1380* families (Lifshitz-Ziv et al., 2018).

IS*Ecp1* has been identified to increase the expression of ESBL genes via a promoter in the upstream region of these genes (Vandecraen et al., 2017). The MGEs are widely prevalent and involve ARGs spread among various environments (Tamang et al., 2013; Jakobsen et al., 2015; Ben Said et al., 2016). The spread of *bla*_CTX-M_ genes was enhanced by IS*Ecp1* element. It is linked to many *bla*_CTX-M_ genes such as *bla*_CTX-M-24_, *bla*_CTX-M-14_, *bla*_CTX-M-79,_ and *bla*_CTX-M-22_ (Tian et al., 2011). Recent studies have found that IS*Ecp1* and IS*26* are the most common structures in the *bla*_CTX-M-55_ flanking environment, with IS*Ecp1*-carrying *bla*_CTX-M-55_ being more prevalent but decreasing year by year in humans and IS*26*-carrying increasing year by year in animals and food (Yang et al., 2023). Previously, in food animals, IS*Ecp1* and IS*903* were observed upstream and downstream to the *bla*_CTX-M-14_ gene while IS*26* transposase was found upstream of the *bla*_CTX-M-1_ gene flanking a partially truncated IS*Ecp1* (Jouini et al., 2007). In the Indian urban aquatic environment, plasmid-mediated gene transmission between genera was observed through HGT; a genetic environment of *bla*_CTX-M-15_ is shown in Figure 2B (Singh et al., 2018).

Rapid HGT in clinical isolates containing *bla*_CTX-M-55_ with four different genetic contexts harboring upstream IS*Ecp1*, truncation by IS*26* and IS*1294*, and downstream orf477 and IS*903* is shown in Figure 2C (Hu et al., 2018). Similar findings have been recorded from blood stream infections in elderly patients, where *bla*_CTX-M_ is linked with IS*Ecp1*, IS*26*, and IS*903* elements with particular special sequences of region V (80 bp), Y(42 bp), and W(48 bp) for IS*Ecp1* (Xiao et al., 2019), as previously described (Eckert et al., 2006; Jouini et al., 2007). Plasmid-mediated dissemination of the *bla*_CTX-M-1_ gene from food-producing animals and healthy human links with the IS*Ecp1* IS*5* and IS*26* elements while the *bla*_TEM_ gene associates with Tn*2* and IS*26* (Figure 2E) (Wang et al., 2015). Recently, high conjugational frequency showed ΔIS*26*-*bla*_CTX-M-14_-ΔIS*903B* transmission among *E. coli* clones and plasmids in pig farm anaerobic effluents (Tian et al., 2022).

Conjugational transfer is a well-known mechanism of HGT between organisms. The transferability of ESBL genes via conjugational experiments has been well documented (Tamang et al., 2013; Mobasseri et al., 2019; Palmeira et al., 2020). Interestingly, one human isolate was found to be a common ST3891 clone carrying *bla*_CTX-M-1_ and *bla*_CTX-M-61_ genes and was also shown in cattle isolated on the same farm. Furthermore, *E. coli* isolates derived from humans, cattle and pigs were found to carry identical *bla*_CTX-M-1_ and *bla*_CTX-M-61_ genes but exhibited distinct STs. This observation may indicate HGT of ARGs between these populations (Dahms et al., 2015). Moreover, ARGs such as *bla*_CTX-M-1_, *bla*_CTX-M-3_, *bla*_CTX-M-9_, *bla*_CTX-M-14_, *bla*_CTX-M-15_, *bla*_TEM-49_, and *bla*_SHV-2_ in WWTP effluents are transferrable in conjugational experiments (Korzeniewska and Harnisz, 2013b; Ben Said et al., 2016), while the *bla*_CTX-M-1_ conjugational transfer was related to the acquisition of IncI1- and IncN-type plasmids (Ben Said et al., 2016). It shows a high probability of HGT between bacteria in sewage and environmental settings. Likewise, different plasmid types such as IncI1 and IncK containing ESBL genes are pooled into farm animals and humans (De Been et al., 2014). Therefore, HGT needs urgent attention and can be reduced through intervention measures, legislative monitoring programs, and minimizing reservoirs or contaminants.

**Figure 3:**
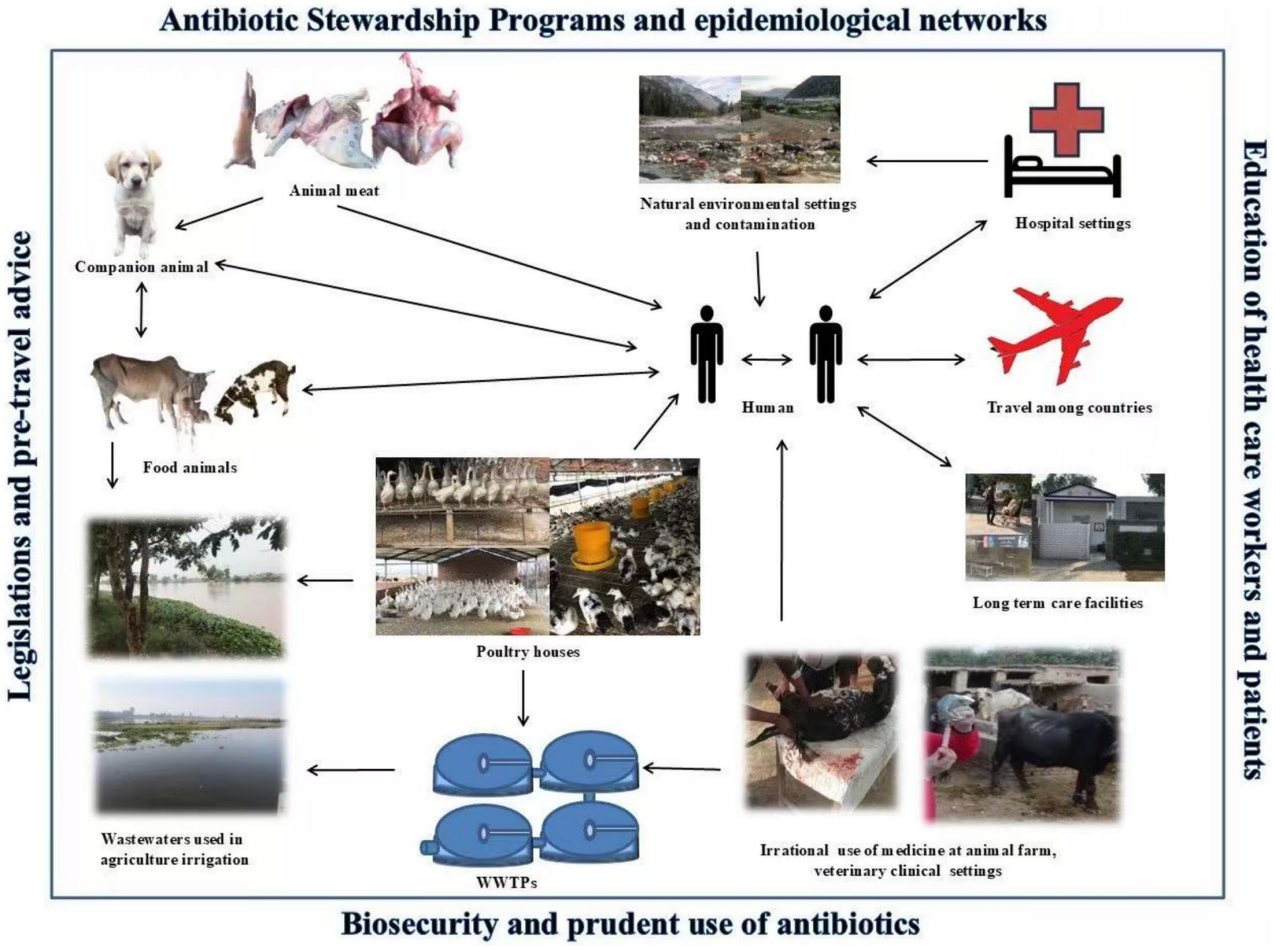
Illustrations of ESBL-PE spread routes in the natural environment. Potential transmission routes of ESBL-PE are indicated by arrows among reservoirs such as hospitals, long-term care facilities, livestock farms, meat, companion animals, travels, WWTPs, water resources, the agricultural field, and humans. The transmission of ESBL-PE in the natural environment can be a hazard to public health. Finally, interventions are given in the outer box which are required to control the AMR from a “One Health” perspective.

Control procedures can be based on the identification of resistant *Enterobacteriaceae* reservoirs (Figure 3). This includes screening and elucidation of ESBL-PE spread routes such as water, air, soil, farm manure, FWs, livestock, poultry, and wildlife (Guenther et al., 2011; Dahms et al., 2015; Dohmen et al., 2017; Wang et al., 2017). In addition to the appropriate use of antibiotics (Mobasseri et al., 2019), disinfection of hospital sewage (Korzeniewska and Harnisz, 2013b) is also recommended. Urinary catheters are a big source of ESBL-PE; direct contact with contaminated instruments can act as a vehicle for HGT. Therefore, it is important to disinfect, sterilize, and properly design instruments to monitor HGT (Al-Jamei et al., 2019). Minimum procedures required for infection control in hospitals should be mandatory, in accordance with WHO guidelines (World Health Organization, 2019). The installation of wastewater treatment facilities in hospitals could be carried out (Adekanmbi et al., 2020) to reduce the introduction of MDR bacteria and ESBL genes in the environment through hospital settings.

Other potential factors may include understanding the transfer of ARB at the livestock interface and other reservoirs (Lee et al., 2020). WWTPs emit ARB into the air, and bio-aerosol emissions can be reduced by covering aeration tanks and grit chambers to control environmental contamination (Korzeniewska and Harnisz, 2013b).

## 11 Prevention and control of ESBL-PE

### 11.1 General procedures to control ESBL-PE

The prevalence of ESBL-PE is growing; therefore, management, specific hygiene measures, and optimal use of antibiotics should be implemented. There is a pressing need to develop approaches to determine the variability of AMR and trends in propagation mechanisms between multiple reservoirs (Figure 3). Training of doctors/staff in ASPs, communication procedures, hygiene, patient information, related management tools (Gillespie et al., 2013; Miclot et al., 2015; Aldrazi et al., 2020; Di Gennaro et al., 2020), excreta management, and strict implementation of rules (NGuyen T. T. H. et al., 2019) are necessary to prevent and control infections involving ESBLs. Studies on the dynamics of transmission, such as surgical site infections (Moremi et al., 2018), and epidemiological networks are also needed between hospital and community settings (Miclot et al., 2015). General practitioners can provide a toolkit that includes a list of infection prevention guidelines, infectious disease physician contacts, treatment procedures, general practitioners, and patient leaflets (Zucconi et al., 2018). Therefore, the spread of ESBL-PE can be measured and reduced to a minimum through active surveillance systems and infection control programs.

Global travel contributes to the spread of ESBL-PE among countries/continents. Active reviews and administrative segregation at the time of admission to medical services for patients who have travelled to Asia, especially with a 6-month history of travel in Southeast Asia and India, should be required (Lübbert et al., 2015; Raffelsberger et al., 2023). Pre-trip advice and awareness should be provided for those who have visited high-risk countries. That can include health safety (Tokinobu et al., 2020), personal hand hygiene procedures (Meurs et al., 2019; Maataoui et al., 2019), and guidelines on antibiotic use during travel (Angelin et al., 2015; Otter et al., 2019; Maataoui et al., 2019). Clinicians need to be careful about ESBL-PE acquired during patient handling. Risk-based monitoring should be carried out for both regular and long-term business visitors (Mizuno et al., 2016). Travel patients should be isolated after rapid recognition of ESBL-PE colonization to prevent further spread of the resistant organism (Lübbert et al., 2015). Thus, effective prevention can be achieved with a better understanding of the dissemination pathways of ESBL-PE in the natural environment (Figure 3).

Hygienic measures such as using specific work clothing, washing hands, taking baths at the end of a work shift, protecting any cuts or lesions on the skin, and wearing a respirator mask could be implemented (Kraemer et al., 2017). There is an urgent need for device, multi-faceted, multi-sectoral, and workable collaboration among stakeholders to contain the spread of ESBL-PE (Founou et al., 2019). Educating FWs, poultry producers, and sellers requires the promotion of hygiene and the prudent use of antibiotics in the poultry production system (Aworh et al., 2019). Finally, effects of municipal wastewater effluent on the spread of AMR in the aquatic environment can be reduced through the application of new treatment technologies, such as silver-based, mesoporous silica-based, titanium dioxide-based, chitosan-based, carbon-based, and clay-based nanomaterials (Ojemaye et al., 2020). The economical and biocompatible ionic liquid choline dihydrogen phosphate has shown to be a promising choice for the elimination of antibiotics from swine effluents (Álvarez et al., 2016). High temperatures in composted pig manure piles can reduce some MGEs such as Tn*3*, IS*Ecp1*, IS*613*, IS*256*, *tnpA-5*, and *tnpA-6* (Wu N. et al., 2020). In general, we are supposed to change our course of action towards a more prudent use of antibiotics. The alternative is to design new therapeutic drug molecules or to make improvements to existing antibiotics such as Halicin via modern machine learning approaches (Stokes et al., 2020). However, ensuring the availability of new drugs in the market is a harder task. One of the solutions is to reintroduce vulnerable flora as probiotics (Imperial and Ibana, 2016). Commensal bacteria can serve as beneficial partners, but strategies are needed to help them outcompete ARB.

### 11.2 Antimicrobial utilization and efficacy of ASPs

The environment plays an important role in elevating selective pressure; for example, the overuse of antimicrobials in agricultural, human, and veterinary settings facilitates bacteria to gain novel mutations essential for survival (Udikovic-Kolic et al., 2014; Berendonk et al., 2015; Woolhouse et al., 2015). Studies have highlighted the link between antibiotic use and AMR within human beings or veterinary settings (Karkaba et al., 2017; Kwok et al., 2020). Thus, there is an urgent need to clarify the role of the clinical laboratory in success of the implementation of ASP to control community infections (Gambrah et al., 2021). For instance, a study in pastoralist out-patients recorded high-resistance ampicillin and 51.6% resistance to amoxicillin-clavulanic acid (Stanley et al., 2018), which highlights the importance of lab testing to improve the precision of antimicrobial therapy. Restricted use of antimicrobials such as cephalosporins has resulted in reduced ESBL usage from 29.5 to 9.5% in hospitals (Kurita et al., 2019). Patients show improved prognosis if they received the right therapy as per laboratory recommendations, as compared to patients who received inappropriate therapy (Fourie et al., 2018). The Ministry of Health, Malaysia, has approved 97 antimicrobials for humans and animals. Alarmingly, these antibiotics, including 3rd and 4th generation cephalosporins, are used in animals as well as in human prophylaxis and therapeutics (Mobasseri et al., 2019). Worryingly, large quantities of antimicrobials/medicines are discarded or unused each year, and most antibiotics used in humans and animals are excreted into the environment through urine and feces, which requires appropriate disposal to reduce the unwanted environmental as well as public health hazards attributed by pharmaceuticals (Zhang et al., 2015; Vatovec et al., 2021).

ASPs are appropriate for patient safety and efficiently reducing adverse drug effects and MDR (Bauer et al., 2019). Although an 100% solution to AMR is impossible, the devoted involvement of the healthcare system is required for the ultimate solution of the problem. The US Center for Disease Control and Prevention (CDC) analyzed effective ASPs in hospitals and recognized core elements for successful ASPs in 2014, which were further updated in 2019. These are hospital leadership commitment, accountability, drug expertise, action, tracking of antibiotic prescription, regular reporting of antibiotic use, resistance to HWs, educating doctors, nurses, and patients about AMR, prescription improvements, and interventions (CDC, 2020). Monitoring of ASPs is important, as results among institutions varied because of the difference in management and control systems. For instance, ASPs have been implemented in Spanish hospitals, and the Spanish Society of Clinical Microbiology and Infectious Diseases has defined the objectives of ASPs along with recommendations and execution (Rodríguez-Baño et al., 2012). As a result, a reduction in rates of UTIs was observed at the hospital level (Esteve-Palau et al., 2018). Further, imposing ASPs in primary health care centers is improving the use of antibiotics and subsequently causing a significant reduction in infections (Peñalva et al., 2020). In addition, education and monitoring of nurses improve the efficacy of ASPs (Gillespie et al., 2013). In Brazil, ASP-sparing *β*-lactam antibiotics was applied: results are promising, as overall antibiotic expenditure decreased by 53.6% after interventions (Zequinao et al., 2020). Overall, ASPs are effectively controlling and decreasing the use of antibiotics/antimicrobials by intervening on antibiotic prescriptions (Bolla et al., 2020; Velasco-Arnaiz et al., 2020). Therefore, the application of ASPs is encouraging and hereby recommended for its wider application from WHO.

## 12 Conclusion

This review updates the prevalence of plasmids in ESBL-PE across the three “One Health” compartments (human, animal, and environment). Key plasmid incompatibility groups include IncF (FIA, FIB, FIC, FI, FII), IncHI (HI1, HI2), IncI1, IncY, IncX (X, X1, X4), IncN, IncA/C, and others (IncB, IncO, IncK, IncZ). ESBL-PE is widely observed in reservoirs such as hospitals, wastewater, WWTPs, agriculture settings, international travel, and companion-, farm-, and food-animals, posing a threat to public health. IS*Ecp1* is present upstream and orf477 downstream to *bla*_CTX-M_ genes with other MGEs, such as IS*26* and IS*903*, playing important roles in the capturing, expression, and mobilization of *bla*_CTX-M_ genes. The *bla*_CTX-M-15_ gene is most prevalent in multiple reservoirs. The HGT is a significant method of gene transfer that is difficult to control. MGEs such as Tn*3* or IS*1380* families and plasmids like IncI1, IncK, and IncN are involved in HGT. The spread of ESBL-PE can be reduced via interventions such as WWTPs facilities in hospitals, knowledge of spread routes, ASPs, and implementation of WHO infection control guidelines in hospitals. Laboratory recommendations of antibiotics should be used in human and veterinary clinics to avoid the overuse of antibiotics. The rise in ESBL-PE is largely due to the misuse of antibiotics in human and veterinary clinics. Policymakers should focus on the rising threat of ESBL-PE and timely implementation of policies as the choice of antibiotics becomes limited.

Competitive omission with probiotics, new drug development, and massive vaccination are future options to decrease ESBL-PE. Therefore, we require exploring the mechanisms involved in the colonization of ESBL-PE. Still, some diverse environments such as schools, offices, local buildings, livestock and poultry farms, and municipal settings need to be investigated for the prevalence of ESBL-PE.
